# Morusin induces osteogenic differentiation of bone marrow mesenchymal stem cells by canonical Wnt/β-catenin pathway and prevents bone loss in an ovariectomized rat model

**DOI:** 10.1186/s13287-021-02239-3

**Published:** 2021-03-12

**Authors:** Ming Chen, Hui Han, Siqi Zhou, Yinxian Wen, Liaobin Chen

**Affiliations:** 1 Department of Joint Surgery and Sports medicine, Zhongnan Hospital of Wuhan University, Wuhan, 430071 China; 2 Hubei Provincial Key Laboratory of Developmentally Originated Disease, Wuhan, 430071 China; 3 Department of Orthopedics Department, Renmin Hospital of Wuhan University, Wuhan, 430060 China

**Keywords:** *Morusin*, BMSCs, Wnt/β-catenin, Osteoporosis

## Abstract

**Background:**

Osteoporosis (OP) is a metabolic bone disease due to the imbalance of osteogenesis and bone resorption, in which, bone marrow mesenchymal stem cells (BMSCs) have a significant effect as the seed cells. Recent research has shown the function of *Morusin* on inhibiting osteoclast differentiation in vitro. However, whether *Morusin* can regulate the osteogenic differentiation in addition to the proliferation of BMSCs remains unclear.

**Methods:**

BMSCs were isolated from 4-week-old Wistar rats and then treated with different concentrations of *Morusin* for 3, 5, 7, and 14 days. The proliferation of BMSCs was detected by MTT assay. The effect of *Morusin* on osteogenic differentiation of BMSCs was detected by RT-qPCR, Western blotting, ALP, and Alizarin Red staining. The effect of *Morusin* on Wnt/β-catenin signaling pathway was analyzed by RT-qPCR, Western blotting, and immunofluorescence. Finally, in the ovariectomy-induced osteoporosis model, the anti-osteoporosis activity of *Morusin* was determined by micro-CT, HE, and immunohistochemistry.

**Results:**

The results showed the function of 2.5–10 μM *Morusin* in the promotion of the proliferation in addition to osteogenic differentiation of BMSCs. Moreover, it also has an impact in activating the Wnt/β-catenin signaling pathway via inhibition of β-catenin phosphorylation as well as promotion of its nuclear translocation. Upon Dickkopf-related protein-1 (DKK-1, an inhibitor of the Wnt/β-catenin signaling pathway) was added to the *Morusin*, *Morusin* had a decreased stimulatory osteogenic effect on BMSCs. Finally, in the rat OP model, we found that *Morusin* could also exert anti-osteoporosis activity in vivo.

**Conclusions:**

This study indicates the ability of *Morusin* in the promotion of osteogenic differentiation of BMSCs via the activation of Wnt/β-catenin signaling pathway and also shows the potential of *Morusin* to be an agent for osteoporosis treatment.

## Introduction

Osteoporosis (OP), a highly prevalent metabolic bone disease, is featured with reduced bone mineral content in addition to structural degeneration of the skeletal system [1, 2]. Generally speaking, OP is a dynamic pathological state resulted from the altered homeostasis between bone resorption of osteoclasts and bone formation of osteoblast [3]. Bone marrow mesenchymal stem cells (BMSCs), considered as pluripotent mesenchymal cells, possess the ability of multilineage differentiation, i.e., the tendency to differentiate into cells of varied categories, such as osteoclasts, chondrocytes, osteoblasts, myocytes, and hepatocytes [4]. To date, there are increasing evidences that the inhibition of osteoblastic activity and differentiation is mainly due to the reduced proliferative potential of BMSCs, especially in the elderly [5–7]. Thus, promoting the osteogenesis of BMSCs may be a promising strategy for OP treatment.

The Wnt/β-catenin signaling pathway is a significant classical pathway in osteogenic differentiation of BMSCs [8]. As a member of the secretory lipid modification signaling glycoproteins family, Wnt is caused to bind to the receptor mainly by palmitoylation. The secreted Wnts (such as Wnt3a) would activate the typical Wnt/β-catenin signaling pathway, when it binds to low density lipoprotein receptor- associated protein 5 (LRP5) or LRP6 and Frizzled transmembrane receptors [9, 10]. Then, a series of cascade reactions lead to cytosolic instability of β-catenin, which then migrates to the nuclei and stimulates osteogenic gene transcription. According to the previous reports, the TLR4 deficiency in knockout mice could promote fracture healing and increase bone mass by way of activating the Wnt/β-catenin signaling pathway [11]. Another study revealed that vasoactive intestinal peptide (VIP) promoted the BMSCs osteogenesis in addition to the angiogenesis differentiation in vitro through activating the Wnt/β-catenin signaling pathway, and advanced bone recovery in vivo [12]. All these evidences suggest Wnt/β-catenin signaling pathways are vital for bone mass maintenance, osteogenic differentiation, and promotion of bone repair.

**Fig. 1:**
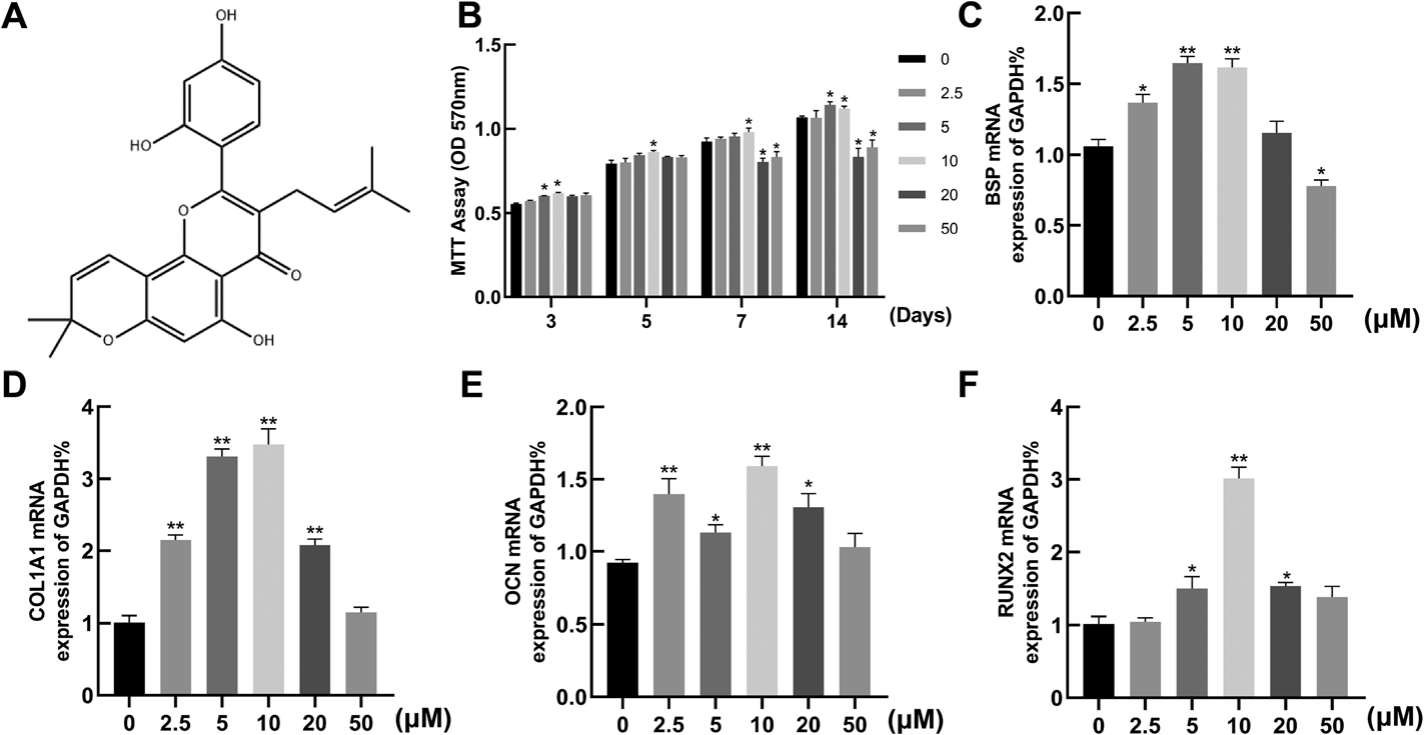
The impacts of *Morusin* on proliferation and osteogenic differentiation of BMSCs. **a** The chemical structure of *Morusin*. **b** Detection of the proliferation toxicity of *Morusin* on BMSCs via MTT assay. BMSCs were cultured in osteogenic growth medium (OGM) with diverse concentrations of *Morusin* for 3, 5, 7, and 14 days. **c**–**f** mRNA level of marker genes for osteogenesis. Quantitative real-time PCR analysis of gene expression of osteogenic differentiation markers. We incubated BMSCs with *Morusin* of diverse concentrations (0–50 μM) for 3 days to observe the impact of *Morusin* on osteogenic differentiation. BMSCs, bone marrow mesenchymal stem cells; RUNX2, runt-related transcription factor 2; COL1A1, collagen type I alpha 1; BSP, bone sialoprotein; OCN, osteocalcin. We repeated the independent experiment for three times, with similar results achieved each time. Means ± SEM. *P < 0.05 and **P < 0.01 in comparison to the control group

*Morusin* (Fig. 1a) is one of the main active substances isolated from mulberry bark, which was proved to have anti-tumor, anti-inflammatory, and antifungal activities [13–15]. Combined with previous researches, we found that *Morusin* could inhibit the progression of human osteosarcoma by means of the phosphatidylinositol 3′-kinase(PI3K)-Akt signaling pathway [16]. Moreover, its effect on bone metabolism is gradually revealed. A recent study identified a novel method for the synthesis of *Morusin* and evaluated the negative regulation of *Morusin* in the osteoclast differentiation in vitro [17]. However, its effect on BMSCs osteogenic differentiation and its possible molecular mechanisms have not been fully illustrated. Herein, we examined the impact and underlying molecular mechanism of *Morusin* in OP, with the purpose of determining whether *Morusin* would be a potential candidate for the OP treatment.

## Materials and methods

### Chemicals and reagents

We allowed *Morusin*, purchased from MedChemExpress (MCE, New Jersey, USA), to dissolve in dimethylsulfoxide (DMSO) and dilute in cell culture solution to make the DMSO content less than 0.1% of the total amount. We procured the penicillin/streptomycin, Trizol, and reverse transcription kit from Thermo Fisher Scientific (Waltham, USA). We ordered the α-Minimum Essential Medium (α-MEM) and Fetal Bovine Serum (FBS) from HyClone Co. (Logan, USA). Rat Dickkopf-related protein 1 (DKK1) was obtained from R&D Systems (Waltham, USA) [18]. The antibody for runt-related transcription factor 2 (RUNX2, sc-390351) was ordered from Santa Cruz Biotechnology Co. (Dallas, USA). The collagen type I alpha 1(COL1A1, A16891), phosphorylated β-catenin (p-β-catenin, AP0579), and β-catenin (A19657) antibody was obtained from ABclonal Technology Co., Ltd. (Wuhan, China). The alizarin red staining kit and alkaline phosphatase chromogenic kit was ordered from Beyotime Co., Ltd. (Shanghai, China). The synthesis of all primers was performed by Tianyihuiyuan Biotech Co., Ltd. (Wuhan, China). As to other reagents, we selected the ones of analytical grade for use in experiments.

### Isolation, culture, differentiation, and treatment of BMSCs

BMSCs were derived from specific pathogen-free (SPF) Wistar rats after isoflurane anesthesia through femoral irrigation in this study. The schemes for isolation, culture, and osteogenic differentiation of BMSCs followed previous researches [19], and the osteogenic growth medium (OGM) included α-MEM, 10–20%FBS, and 100 IU/ml penicillin/streptomycin. Then, treatment of the cells was conducted with *Morusin* at diverse concentrations (0, 2.5, 5, 10, 20, and 50 μM) in the osteogenic differentiation medium (ODM, α-MEM with 0.1 μM hexadecadrol, 10–20% FBS, 50 mM l-ascorbic acid-2-phosphate, 100 IU/ml penicillin streptomycin combination, and 10 mM β-glycerophosphate) to determine the optimal concentration of *Morusin* for increasing proliferation and osteogenic differentiation of BMSCs. Next, 500 ng/ml of Dickkopf-related protein-1 (DKK-1, an inhibitor of the Wnt/β-catenin signaling pathway) was applied for investigating the involvement of Wnt/β-catenin pathway in *Morusin* promoting the differentiation and proliferation of BMSCs [20].

### BMSC proliferation assay

To observe the impact of diverse *Morusin* concentrations in BMSC proliferation, cells were inoculated in 96-well plates (1 × 10^4^ per well) and MTT assay was performed. Next, we added *Morusin* (0, 2.5, 5, 10, 20, and 50 μM) to cells in OGM on 3, 5, 7, and 14 days. At each time point, we added 20 μL MTT (5 mg/mL) reagents to each well for incubating the cells at 37 °C for 2 h. The viability of BMSCs was evaluated by a microplate reader (Thermo Fisher Scientific, USA) through the measurement of the absorbance at 570 nm after the formazan crystals were dissolved in DMSO.

### ALP staining and alizarin red staining(ARS)

BMSCs were plated on 12-well plates at a density of 1 × 10^4^ per well and differentiated by ODM for 3 or 10 days, respectively. After fixing the cells with 4% paraformaldehyde for 30 min, we used PBS for washing the cells for three times, and then, we adopted alkaline phosphatase chromogenic kit to incubate the cells at room temperature in darkness for 1 h for staining. Finally, we employed an inverted microscope (Olympus, Japan) for observing and photographing the staining results.

ARS was used to detect mineral deposition after BMSCs induced differentiation. We inoculated the cells on 6-well plates with a density of 1 × 10^6^ per well and differentiated them by ODM for 14 days. Then, the cells were fixed and washed as aforementioned. After that, we added Alizarin Red staining solution to each well of the 6-well plate for 15 min. They were then rinsed again with PBS and the calcium salt deposition were observed via an inverted microscope (Olympus, Japan).

### Total RNA extraction and quantitative real-time PCR (RT-qPCR)

After osteogenesis induction, we used PBS for washing BMSCs before adding Trizol reagent to isolate total RNA from BMSCs. We determined the purity and concentration of the isolated RNA via a Nano-Drop-2000 nucleic acid analyzer. Then we adopted a reverse transcription kit for conversion of the total RNA into cDNA. We evaluated the expression levels of mRNA by one-step RT-qPCR with thermal Cycler (ABI Step One, USA). Then, we applied the 2^−ΔΔCt^ method for analysis of the target genes in terms of the expression level before we normalized all values to the expression level of glyceraldehyde phosphate dehydrogenase (GAPDH) mRNA. Table 1 described the primer sequences of every group of genes applied in this experiment.

**Table 1:** The primer sequences of genes in this experiment

Target genes	Forward primer	Reverse primer
COL1A1	TGTCGTTCAACGGCACAG	TGTGGTAGACTCCACGACA
RUNX2	GAGCGTTCAACGGCACAG	GACAGTAGACTCCACGACA
BSP	CGTCTCCATGGTGGATTATG	GGATGTAGTTCTGCTCATGG
OCN	GTCAGACTACAACATCCAGAAG	CGAGTATCTTCCTGTTTGACC
β-catenin	GCCATCACCACGCTGCATAATCT	GGCAGTCTGTCGTAATAGCCAAGAA
Dvl-1	CCTTCCATCCAAATGTTGCCAGTA	GGGCAGCCTCATCACGGTTT
LEF-1	CACACAACTGGCATCCCTCATC	GCTCCTGTTCCTTTCTCTCTGTTCGT
GAPDH	GGGTGTGAACCACGAGAAAT	ACTGTGGTCATGAGCCCTTC

*COL1A1* collagen type I alpha 1, *RUNX2* runt-related transcription factor 2, *BSP* bone sialoprotein, *OCN* osteocalcin, *Dvl-1* dishevelled-1, *LEF-1* lymphoid enhancing factor-1, *GAPDH* glyceraldehyde phosphate dehydrogenase

### Western blot (WB)

We used precooled PBS for washing the cells for thrice. We, then, adopted 150 μl RIPA Lysis Buffer and 1 mM protease inhibitor for lysing the cells for 25 min on ice to extract the total protein. We separated the same amounts of proteins using 10% or 12.5% sodium dodecyl sulfate polyacrylamide gel electrophoresis (SDS-PAGE) before transferring them to polyvinylidene difluoride (PVDF) membranes. 3% bovine serum albumin (BSA) or 5% skim milk was employed for blocking the membranes for 60 min. Then, we adopted primary antibodies for membrane incubation at 4 °C for 16 h, including GAPDH (1:2000 dilution), RUNX2 (1:300 dilution), p-β-catenin (1:1000 dilution), β-catenin (1:1000 dilution), and COL1A1(1:1000 dilution). TBST was applied in membrane washing for 3 times (5 min each) and a fluorescence-conjugated secondary antibody (1:5000 dilution) was employed for membrane incubation for 1 h. Finally, enhanced chemofluorescence reagents were used for visualization of immunoreactive protein signals.

### Immunofluorescence analysis

Precooled PBS was used for washing BMSCs cultured in 6-well plates for 3 times. Then, we adopted 4% paraformaldehyde to fix BMSCs at room temperature for 15 min. After we washed the cells for twice with PBS, we implemented 0.5% Triton X-100 to permeabilize the cells for 15 min and employed 3%BSA to block the cells at room temperature for 40 min. We washed the blocked BMSCs before their overnight incubation at 4 °C for 12 h with the primary antibodies of COL1A1(1:200 dilution), RUNX2(1:50 dilution), and β-catenin (1:200 dilution) protein. Again, we used PBS for washing the cells for 3 times before their incubation with a fluorescein-linked secondary antibody (1:100 dilution) for 40 min at room temperature. Then, we stained the nuclei with 4′,6-diamidino-2-phenylindole (DAPI, Thermo Fisher Scientific, Waltham, USA) for 5 min. Finally, we observed the cellular samples through a fluorescence microscope (Nikon, Japan).

### Ovariectomized (OVX) rat model

We purchased all specific pathogen-free Wistar rats [female, aged 16 weeks and weighted 230–360 g, NO.2020-0018, license number: SCXK (Hubei), certification number: 42000600038191] from the Experimental Center of the Hubei Medical Scientific Academy (Wuhan, China). All animal experiments were conducted in the Center for Animal Experiment of Wuhan University (Wuhan, China), and all protocols were authorized by the Animal Welfare Committee in Wuhan University (License number: 14016).

Firstly, we randomly classified 15 female Wistar rats into three groups: Sham operated group, OVX group, and OVX *& Morusin* (40 mg/kg) group [21]. Both the OVX group and the OVX & *Morusin* group received ovariectomies including bilateral ovaries, ovarian capsule, partial fallopian tubes, and fed with a calcium-deficient diet (0.1% calcium and 0.77% phosphorus) to induce experimental osteoporosis, whereas the Sham operated group underwent laparotomy without oophorectomy and fed with a standard diet. At 6 weeks following OVX, *Morusin* (40 mg/kg, dissolved by DMSO and then diluted by saline, with a final DMSO concentration of 5%) intragastric administration was administered to rats in the OVX & *Morusin* group every 5 days, while rats in the Sham and OVX group were intragastricly administrated with 5% dimethylsulfoxide (DMSO) in saline. After 4 weeks of *Morusin* treatment, the rats were euthanized and sacrificed; then, we collected the femurs. After that, the left side of femurs was soaked in 4% paraformaldehyde for 2 days at 4 °C for subsequent pathological testing, while the right was applied for micro-CT and other tests.

### Histological and immunohistochemistry analysis

Femurs were fixed overnight with 4% paraformaldehyde before being dehydrated and embedded in paraffin. Then, hematoxylin and eosin (HE) was used for femur section staining to observe bone trabecular area and related bone mass indexes [osteoblasts number/bone perimeter (N.Ob/B.Pm) and osteoblast surfaces/bone surface (Ob.S/BS)]. Then, immunohistochemistry was performed to determine the level of RUNX2 protein in the femur. The dilution of RUNX2 primary antibody was 1:100. An optical microscope (Olympus, Japan) was implemented for the staining result observation and photographing. Then, we analyzed the stained images with image J software (version 6.0) before measurement of the mean optical density (MOD) of 5 random fields in every section for determining the staining intensity.

### Micro-CT scanning

Femurs fixed in 75% alcohol were scanned and analyzed with skyscan1276 μCT system (Bruker, Germany). The sample was placed in a transparent cylindrical groove and secured with paper tape. The scanning mode was set to 0.5-mm filter, 450-V scanning voltage, and 5-μm scanning resolution. After scanning data reconstruction, the region of interest was selected for non-volume-related parameter analysis [trabecular separation (Tb.Sp), trabecular number (Tb.N), bone volume/tissue volume (BV/TV), connectivity density (Conn.Dens.), trabecular thickness (Tb.Th), and structure model index (SMI)] and reconstructed to obtain 3D images [22].

### Data analysis

We indicated all experimental values as mean ± standard error of mean (SEM) of the experimental values (we repeated each experiment for three times). For comparison between the two groups, we performed double-tailed Student *t* test to determine the statistical significance. For comparison between more than two groups, we implemented one-way ANOVA. A *p* < 0.05 showed statistically significant difference.

## Results

### The influence of *Morusin* in the proliferation and osteogenesis differentiation of rat BMSCs in vitro

During BMSC proliferation, *Morusin* at diverse concentrations (0–50 μM) was treated for 3–14 days. Then, we executed MTT assay. As per the results, there was promoted proliferation detected in the osteogenic BMSCs treated with *Morusin* at 2.5, 5, and 10 μM (Fig. 1b) in days 3, 5, 7, and 14, while *Morusin* of 20 μM and 50 μM both showed an inhibitive effect on the proliferation of BMSCs in days 7 and 14. The peak of the promotive effect on the proliferation of BMSCs was observed in the group treated with 10 μM *Morusin*. Then, we further investigated the effects of *Morusin*’s concentration (0–50 μM) on BMSC differentiation and detected the levels of osteogenic marker genes including bone sialoprotein (*Bsp*), *Col1a1*, *Runx2*, and osteocalcin (*Ocn*). *Morusin* was found to promote osteogenic marker genes in terms of the expression at 2.5, 5, and 10 μM, while 10 μM of *Morusin* revealed the strongest effect (Fig. 1c–f). The above results indicated the function of *Morusin* in promoting the differentiation in addition to proliferation of BMSCs at 2.5–10 μM. So, the concentrations of 2.5, 5, and 10 μM were selected for subsequent studies.

### *Morusin* promotes osteogenesis of BMSCs in vitro

**Fig. 2:**
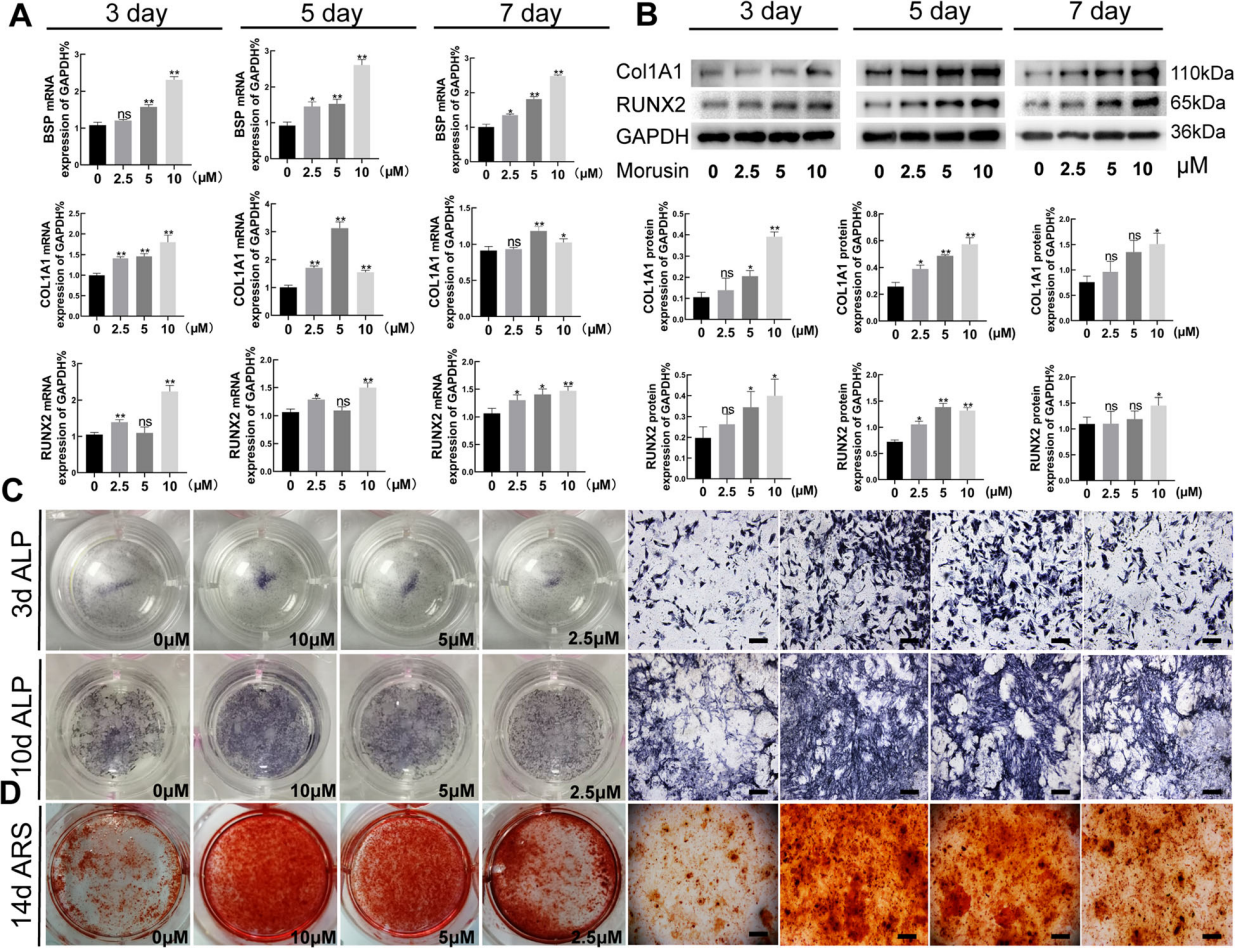
Influences of *Morusin* on osteogenic differentiation of BMSCs. **a** Detection of the expression of osteo-specific genes (*Col1a1*, *Runx2*, and *Bsp*) at days 3, 5, and 7 of osteogenic differentiation via quantitative real-time PCR. We normalized the gene expression levels to GAPDH. **b** Relative protein expression of osteo-specific genes (COL1A1 and RUNX2) at days 3, 5, and 7 of osteogenic differentiation. The protein expression level is the gray value ratio of the target protein to GAPDH. **c** ALP staining of the osteogenic BMSCs. Scale bar, 250 μm. **d** Alizarin red staining of the osteogenic BMSCs. Scale bar, 250 μm. BMSCs, bone marrow mesenchymal stem cells; RUNX2, runt-related transcription factor 2; COL1A1, collagen type I alpha 1; BSP, bone sialoprotein. We repeated the independent experiment for three times. Means ± SEM. *P < 0.05 and **P < 0.01 in comparison to the control group. “ns” refers to no statistical significance compared with the control group

Stimulated mRNAs expression of *Col1a1*, *Runx2*, and *Bsp* was detected in BMSCs disposed with 2.5, 5, and 10 μM of *Morusin* for 3, 5, and 7 days, in comparison to the control group (Fig. 2a). COL1A1 and RUNX2 protein expression was also found increased in osteogenic BMSCs with *Morusin* at days 3, 5, and 7 (Fig. 2b). Further, deeper ALP staining was observed in the osteogenic BMSCs after 3 and 10 days of *Morusin* treatment (Fig. 2c). Meanwhile, more calcium nodules were also caught sight of in the osteogenic BMSCs disposed with *Morusin* after 14 days of differentiation, especially in these treated with 10 μM *Morusin* (Fig. 2d). The above results indicated that *Morusin* could promote the osteogenic differentiation of BMSCs in vitro.

### Wnt/β-catenin pathway participated in the promoted osteogenic differentiation of BMSCs treated with *Morusin* in vitro

**Fig. 3:**
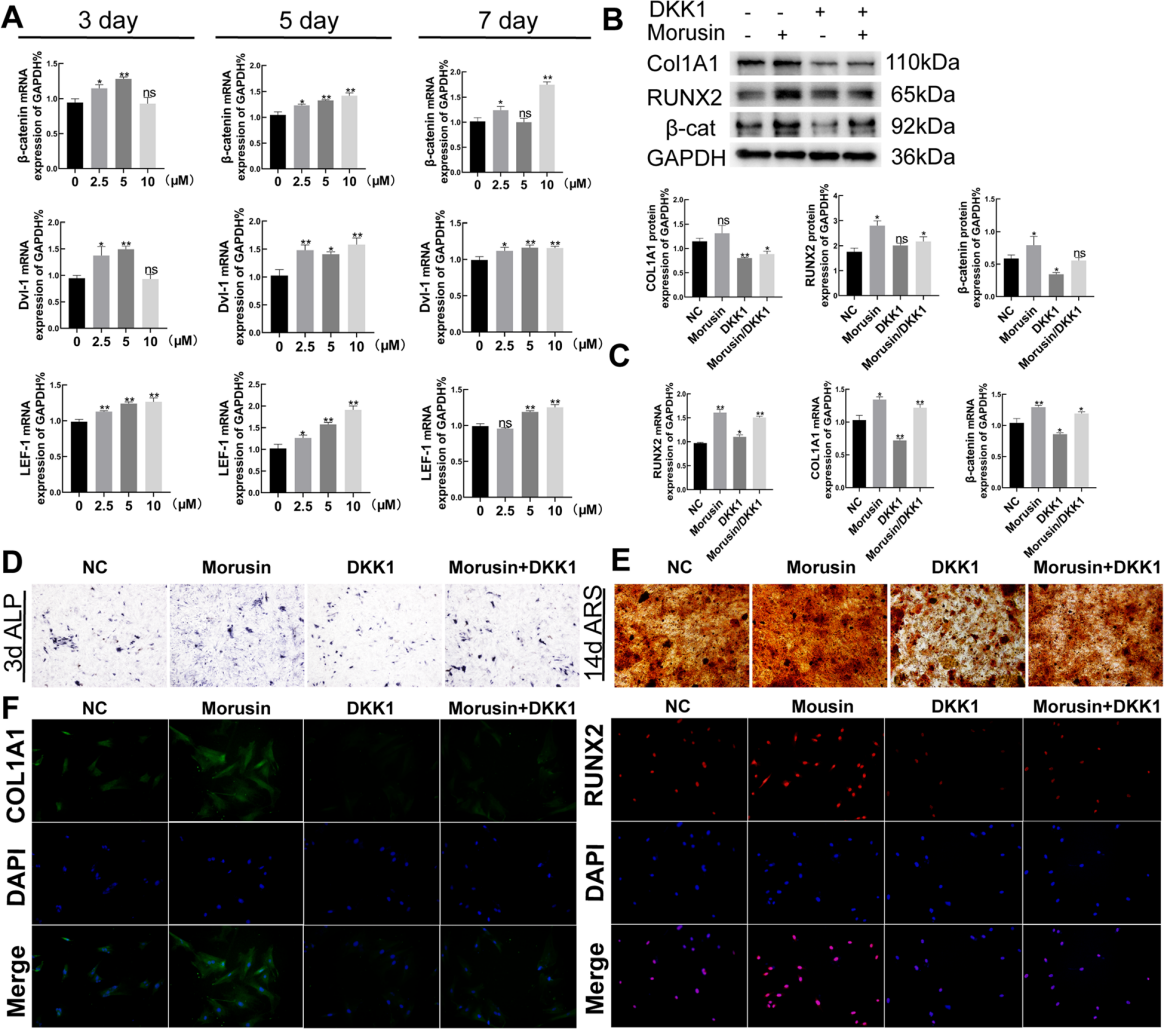
*Morusin* promotes the Wnt/β-catenin signaling pathway in BMSCs. **a** Detection of the expression of target genes in Wnt/β-catenin pathway via quantitative real-time PCR. We normalized the gene expression levels to GAPDH. **b**, **c** DKK-1 partially reversed the pro-expression effect of *Morusin* (10 μM) on *Col1a1*, *Runx2*, and *β-catenin*. The protein expression level is the gray value ratio of the target protein to GAPDH. Gene expression was normalized to GAPDH. **d** ALP staining of the osteogenic BMSCs. BMSCs were treated with *Morusin* (10 μM) and DKK-1(500 ng /ml) for 3 days, fixed and stained with ALP staining. Scale bar, 250 μm. **e** Alizarin red staining of the osteogenic BMSCs. The cells were incubated with *Morusin* (10 μM) and DKK-1(500 ng/ml) for 14 days, and the calcium deposits were recognized via Alizarin red staining. Scale bar, 250 μm. **f** BMSCs immunofluorescence results of RUNX2 and COL1A1. Scale bars, 100 μm. BMSCs, bone marrow mesenchymal stem cells; RUNX2, runt-related transcription factor 2; COL1A1, collagen type I alpha 1; Dvl-1, dishevelled-1; LEF-1, lymphoid enhancing factor-1; NC, negative control; DKK-1, Dickkopf-related protein-1. We repeated the independent experiment for three times. Means ± SEM. *P < 0.05 and **P < 0.01 in comparison to the control group. “ns” refers to no statistical significance in comparison to the control group

Wnt/β-catenin signaling pathway is vital for BMSC differentiation and is a significant pathway for promoting new bone formation, inhibiting bone resorption, and maintaining bone mass [23, 24]. In the current study, increased expression of β-catenin, lymphoid enhancing factor-1(*Lef-1*), and dishevelled-1 (*Dvl-1*) mRNAs were detected in BMSCs treated with *Morusin* (0–10 μM) on the 3rd, 5th, and 7th days of differentiation, respectively (Fig. 3a). Then, further addition of 500 ng/ml DKK-1, as an effective inhibitor of Wnt /β-catenin signaling pathway, attenuated such effects of *Morusin* (10 uM) about the gene expression of *β-catenin*, *Lef-1*, and *Dvl-1* (Fig. 3b, c), which was further confirmed by immunofluorescence assay (Fig. 3f). In addition, DKK-1 treatment attenuated ALP staining and calcium nodule deposition in the osteogenic BMSCs treated with *Morusin* after 3 and 14 days of differentiation, respectively. (Fig. 3d, e). These results suggest the probability of Wnt/β-catenin pathway to take part in osteogenic differentiation of promoting effect of *Morusin* in vitro*.*

### *Morusin* affected phosphorylation of β-catenin and nucleus translocation of β-catenin in vitro

**Fig. 4:**
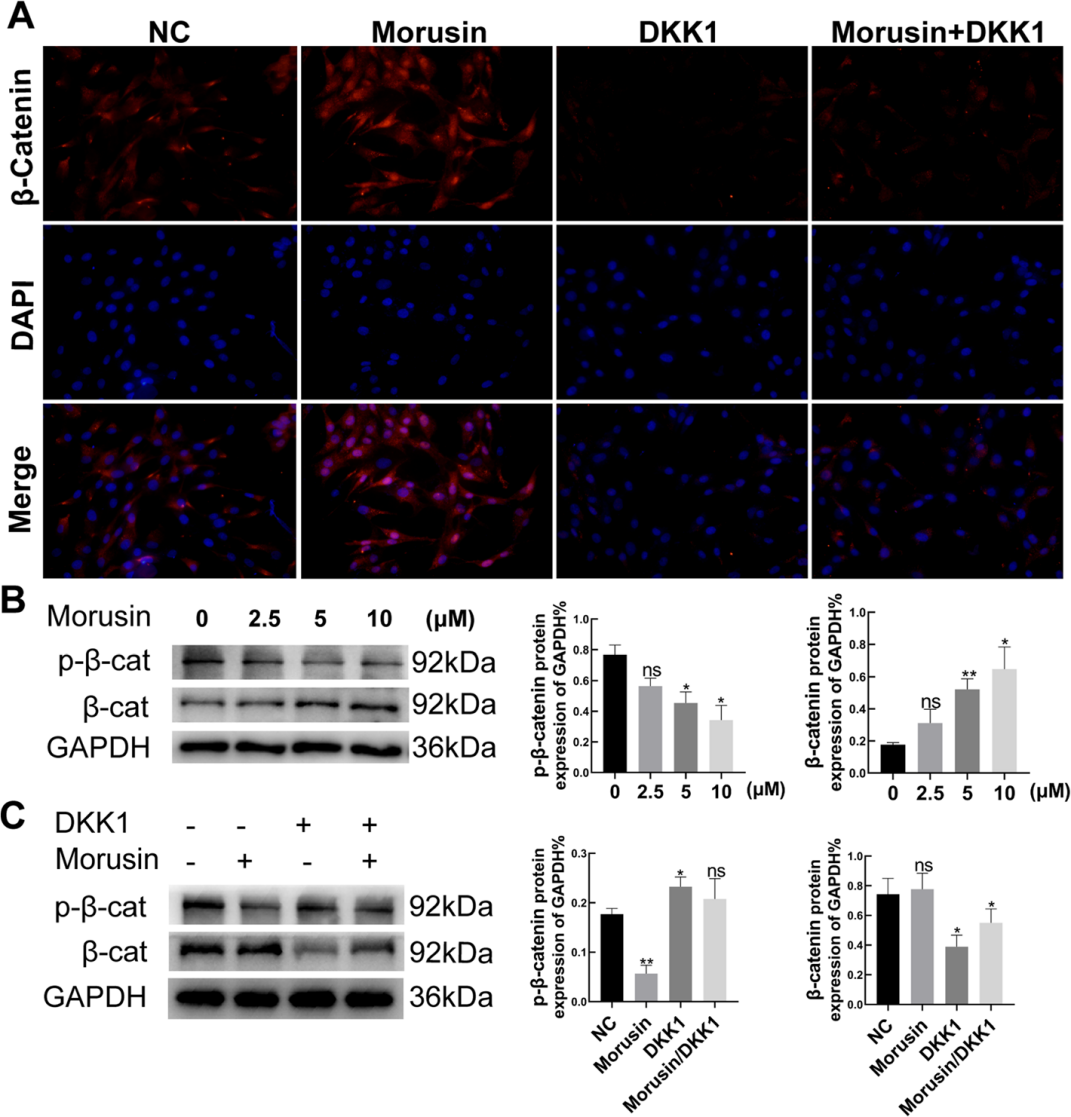
The effect of *Morusin* on phosphorylation and nuclear transport of β-catenin. **a** Immunofluorescence assay of β-catenin in the osteogenic BMSCs. *Morusin* activates nuclear transposition of β-catenin in BMSCs. Scale bars, 100 μm. **b** Total and phosphorylated β-catenin in the osteogenic BMSCs treated with *Morusin* (10 μM) by Western Blotting. The protein expression level is the gray value ratio of the target protein to GAPDH. **c** Total and phosphorylated β-catenin in the osteogenic BMSCs treated with *Morusin* (10 μM) and DKK-1 (500 ng /ml) by Western Blotting. The protein expression level is the gray value ratio of the target protein to GAPDH. BMSCs, bone marrow mesenchymal stem cells; NC, negative control; p-β-catenin, phosphorylated β-catenin. We repeated the independent experiment for three times. Means ± SEM. *P < 0.05 and **P < 0.01 in comparison to the control group. “ns” refers to no statistical significance compared with the control group

When Wnt/β-catenin signaling pathways are activated, they are often accompanied by phosphorylation inhibition and nuclear transport of β-catenin. To further explore *Morusin*’s effect on nuclear transport of β-catenin, we performed immunofluorescence detection of β-catenin. The consequences revealed that *Morusin* (10 μM) heightened the expression of β-catenin (red) in the nuclei, while DKK-1 (500 ng/ml) partially reversed *Morusin*’s effect on β-catenin translocation (Fig. 4a). Further, it was found that *Morusin* (0–10 μM) had the effect in inhibiting the phosphorylation of β-catenin. Namely, with the increase of *Morusin* concentrations, the phosphorylated β-catenin decreased in terms of the protein expression, while total β-catenin increased (Fig. 4b). Afterward, the phosphorylation inhibition of β-catenin by *Morusin* (10 μM) was partially reversed by DKK-1 (500 ng/ml) (Fig. 4c). These results indicated the function of *Morusin* to activate the Wnt/β-catenin signaling pathway via inhibition of β-catenin phosphorylation and promotion of its nucleus translocation.

### *Morusin* attenuated bone loss in OVX rats

**Fig. 5:**
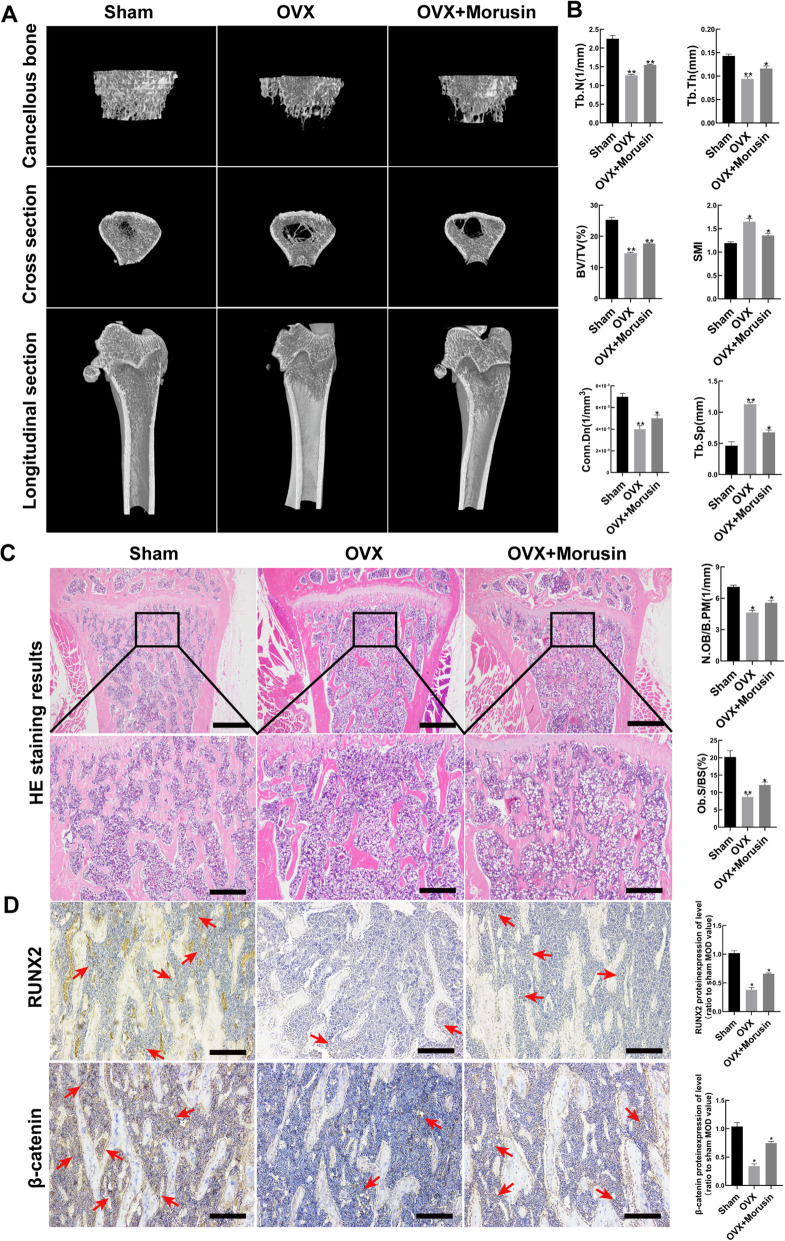
*Morusin* attenuated bone resorption in OVX rats. **a**, **b** Representative three-dimensional reconstruction image and micro-CT analysis of femurs in Sham operated rats, OVX rats, and OVX & *Morusin* group. Scale bar, 500 μm. **c** Representative images of HE staining of femurs in Sham operated rats, OVX rats and OVX & *Morusin* group. Scale bar, 500 μm. Scale bar (enlarged), 250 μm. **d** *RUNX2* and *β-catenin* immunochemical staining of femurs in Sham operated rats, OVX rats, and OVX & *Morusin* group. The red arrows indicate the highly expressed region of *RUNX2* and *β-catenin*. Scale bar, 100 μm. OVX, ovariectomized; BV/TV, bone volume/tissue volume; Tb.N, trabecular number; Tb.Th, trabecular thickness; Tb.Sp, trabecular separation; Conn.Dn, connectivity density; SMI, structure model index; RUNX2, runt-related transcription factor 2; N.Ob/B.Pm, osteoblasts number/bone perimeter; Ob.S/BS, osteoblast surfaces/bone surface. We repeated the independent experiment for three times. Means ± SEM. *P < 0.05 and **P < 0.01 in comparison to the control group

*Morusin* (40 mg/kg) was admitted to the OVX rats to evaluate the effect of *Morusin* on OP. Micro-CT scan results showed that *Morusin* (40 mg/kg) could alleviate the bone loss caused by estrogen deficiency to some extent, which was manifested in increased Tb.N, Tb.Th, BV/TV, and Conn.Dn, while a decrease in Tb.Sp and SMI (Fig. 5a, b). Furthermore, HE staining revealed that in comparison to the OVX group, an increase in the number of bone trabeculae and bone mass indexes (N.Ob/B.Pm and Ob.S/BS) was observed in the OVX & *Morusin* group (Fig. 5c). Similarly, immunohistochemical results also showed that *Morusin* could attenuate the decrease of the expression of *RUNX2* and *β-catenin* in the femur of OVX rats (Fig. 5d). Such results indicated that *Morusin* could attenuate bone loss in OVX rats. Therefore, we believe that *Morusin* would be a potential novel candidate for anti-OP.

## Discussion

Due to the high prevalence of osteoporosis in the elderly, the number of patients is still keeping increasing, and over 60 million of osteoporosis patients were reported in China in 2019 [25]. The severe complication of brittle fracture has a higher disability rate and mortality rate, which threatens people’s quality of life, as well as their physical and mental health to a great extent [26]. How to prevent and treat osteoporosis effectively has become one of the major clinical and experimental issues. Osteogenesis of osteoblasts and bone resorption of osteoclasts is important for the occurrence and development of osteoporosis [27–29]. As shown in numerous previous studies, promoting BMSC osteogenic differentiation could effectively prevent or slow down the occurrence and development of osteoporosis [30–32]. *Morusin*, a monomer component from a widely used traditional Chinese medicine Cortex Mori, was proved to be with bioactivities of anti-tumor, anti-inflammatory, and anti-oxidation [33–35]. Although *Morusin* have been shown to have the function on inhibiting osteoclast differentiation in vitro, there has been no illumination of the function of *Morusin* about BMSC proliferation and osteogenic differentiation [17]. In this study, how *Morusin* affect BMSC proliferation was first examined. 10 μM *Morusin* was the optimum concentration to promote BMSC proliferation and had no obvious cytotoxicity on the basis of the results of the MTT assay. Then, we found that *Morusin* could promote the osteogenic differentiation of BMSCs in the manner of concentration dependence from 2.5 to10 μM. Further, we selected different differentiation time points of BMSCs to verify the role of *Morusin* and found that *Morusin* could promote the expression of osteogenic marker genes at different stages of BMSC differentiation. As an early osteogenic marker, ALP promotes mineralization during osteogenic differentiation. ARS can indicate the number of calcium nodules at the late stage of differentiation [36]. Both of them can directly reflect the effect of osteogenic differentiation. This study showed that *Morusin* obviously raised the ALP activity and caused the formation of calcium nodules to happen in BMSCs. All the above findings revealed that *Morusin* was safe and effective in promoting osteogenic differentiation of BMSCs in vitro*.*

Although *Morusin* has the ability to effectively advance osteogenic differentiation of BMSCs, its underlying mechanism remains unclear. As confirmed by previous studies, Wnt/β-catenin signaling pathway is a great mediator of BMSCs differentiation into osteoblasts [37–39]. Therefore, we studied the function of Wnt/β-catenin signaling pathway in the osteogenesis of BMSCs treated by *Morusin*. Our results showed that *Morusin* could significantly increase genes related to Wnt/β-catenin signaling in terms of the expression (*β-catenin*, *Dvl-1*, and *LEF-1*), while DKK-1 can partially block the promoting effect of *Morusin* on osteogenic differentiation of BMSCs. When Wnt/β-catenin signaling pathway is activated, it can lead to the disintegration of APC, Axin, and GSK3β complex. Then, inhibition of β-catenin phosphorylation raises the levels of β-catenin in the cell and allows β-catenin to enter the nuclei to bind to the transcription factor TCF/LEF, thereby initiating transcription of downstream target genes [40]. Thus, we further examined phosphorylation levels of β-catenin and its nucleus translocation. As per these results, *Morusin* had an impact of inhibition about the phosphorylation of β-catenin but promoted its nucleus translocation. These findings revealed that *Morusin* had the ability in advancing osteogenic differentiation of BMSCs via activating Wnt/β-catenin signaling pathway.

The impact of *Morusin* on bone loss in rats was further investigated. In this research, OVX rats were used as the osteoporosis model, which has been widely regarded as a model with low osteogenic capacity of the BMSCs [37, 41]. Micro-CT, HE staining, and immunohistochemical analysis showed that after *Morusin* treatment, the bone loss of OVX rats was reduced, the number of osteoblasts was increased, and the protein expression of osteogenic marker genes was also increased. These evidences show that *Morusin* can promote osteoblastic proliferation and bone formation in OVX rats, which is consistent with vitro experiment.

## Conclusion

It could be concluded that our current study demonstrated the ability of *Morusin* in promoting the osteogenic differentiation in addition to proliferation of BMSCs via activation of Wnt/β-catenin signaling pathway in vitro, and also presented anti-osteoporosis activity in OVX rats. This study also suggested the potential of *Morusin* to be a therapeutic agent for osteoporosis. Further researches are needed before its clinical application.

## Data Availability

The datasets used and/or analyzed during the current study are available from the corresponding author on reasonable request.

## Abbreviations

BMSCs: Bone marrow mesenchymal stem cells
OP: Osteoporosis
DKK-1: Dickkopf-related protein-1
LRP5: Lipoprotein receptor-associated protein 5
DMSO: Dimethylsulfoxide
OGM: Osteogenic growth medium
ARS: Alizarin red staining
RT-qPCR: Quantitative real-time PCR
GAPDH: Glyceraldehyde phosphate dehydrogenase
COL1A1: Collagen type I alpha 1
RUNX2: Runt-related transcription factor 2
BSP: Bone sialoprotein
OCN: Osteocalcin
Dvl-1: Dishevelled-1
LEF-1: Lymphoid enhancing factor-1
OVX: Ovariectomized
N.Ob/B.Pm: Osteoblasts number/bone perimeter
Ob.S/BS: Osteoblast surfaces/bone surface
Tb.Sp: Trabecular separation
Tb.N: Trabecular number
BV/TV: Bone volume/tissue volume
Conn.Dens: Connectivity density
Tb.Th: Trabecular thickness
SMI: Structure model index
